# A Horrible Traffic

**Published:** 1886-09

**Authors:** 


### Editorial.

### A HORRIBLE TRAFFIC.

Can anyone imagine anything more horrible than the traffic in dead bodies of human beings? We think not, and yet it is a business in Georgia, the proportions of which, if known, would cause a shudder to come over almost every one.

The general impression is that only a few graves are robbed in Georgia for the supply of the medical colleges in the State. This, however, is not true. It is well known, by some at least, that there are a number of bodies shipped to colleges out of the State every year.

There is a great demand for bodies for dissecting purposes in States where the law has made no provision for the colleges. This demand will continue to exist so long as the law remains as at present, and there will always be found men who will undertake to supply the demand. There are several States where the law makes provision for dissecting, and in those States the supply, in nearly every instance, exceeds the demand and grave robbing is unknown.

This fact should influence the people of our State to demand at the hands of the law-makers protection from these ghouls by making provision for our colleges. This, and this alone, will give them the protection they want and deserve.

If the next legislature of this State would make proper provision for our colleges, and at the same time make grave-robbing and the shipping of dead bodies beyond the State a felony, we would hear no more of it. Every physician in Georgia would be interested in seeing such a law enforced, and there would be no longer any field here for agents of medical colleges outside of the State.

We believe the profession can do much towards accomplishing this most desirable result by their influence among the people and the members of the legislature. Will they do it?

